# Reduction of High Levels of Internal Radio-Contamination by Dietary Intervention in Residents of Areas Affected by the Fukushima Daiichi Nuclear Plant Disaster: A Case Series

**DOI:** 10.1371/journal.pone.0100302

**Published:** 2014-06-16

**Authors:** Masaharu Tsubokura, Shigeaki Kato, Shuhei Nomura, Stuart Gilmour, Masahiko Nihei, Yu Sakuma, Tomoyoshi Oikawa, Yukio Kanazawa, Masahiro Kami, Ryugo Hayano

**Affiliations:** 1 Division of Social Communication System for Advanced Clinical Research, Institute of Medical Science, University of Tokyo, Minato-ku, Tokyo, Japan; 2 Department of Radiation Protection, Soma Central Hospital, Soma, Fukushima, Japan; 3 Department of Epidemiology and Biostatistics, School of Public Health, Imperial College London, Norfolk Place, London, United Kingdom; 4 Department of Global Health Policy, Graduate School of Medicine, University of Tokyo, Bunkyo-ku, Tokyo, Japan; 5 Hirata Radioactivity Inspection Center, Hirata Central Hospital, Hirata, Fukushima, Japan; 6 Department of Radiation Protection, Minamisoma Municipal General Hospital, Minamisoma, Fukushima, Japan; 7 Department of Physics, University of Tokyo, Bunkyo-ku, Tokyo, Japan; Kagoshima University Graduate School of Medical and Dental Sciences, Japan

## Abstract

Maintaining low levels of chronic internal contamination among residents in radiation-contaminated areas after a nuclear disaster is a great public health concern. However, the efficacy of reduction measures for individual internal contamination remains unknown. To reduce high levels of internal radiation exposure in a group of individuals exposed through environmental sources, we performed careful dietary intervention with identification of suspected contaminated foods, as part of mass voluntary radiation contamination screenings and counseling program in Minamisoma Municipal General Hospital and Hirata Central Hospital. From a total of 30,622 study participants, only 9 residents displayed internal cesium-137 (Cs-137) levels of more than 50 Bq/kg. The median level of internal Cs-137 contamination in these residents at the initial screening was 4,830 Bq/body (range: 2,130–15,918 Bq/body) and 69.6 Bq/kg (range: 50.7–216.3 Bq/kg). All these residents with high levels of internal contamination consumed homegrown produce without radiation inspection, and often collected mushrooms in the wild or cultivated them on bed-logs in their homes. They were advised to consume distributed food mainly and to refrain from consuming potentially contaminated foods without radiation inspection and local produces under shipment restrictions such as mushrooms, mountain vegetables, and meat of wild life. A few months after the intervention, re-examination of Cs levels revealed remarkable reduction of internal contamination in all residents. Although the levels of internal radiation exposure appear to be minimal amongst most residents in Fukushima, a subset of the population, who unknowingly consumed highly contaminated foodstuffs, experienced high levels of internal contamination. There seem to be similarities in dietary preferences amongst residents with high internal contamination levels, and intervention based on pre- and post-test counseling and dietary advice from medical care providers about risky food intake appears to be a feasible option for changing residents' dietary practices, subsequently resulting in a reduction in Cs internal contamination levels.

## Introduction

Radiation exposure can result in an increased risk of long-term health problems, such as development of malignant tumors, with the risk being strongly related to personal exposure doses [1]. Accordingly, serious health threats have emerged in radiation-contaminated areas after nuclear accidents such as the Chernobyl accident [2], and similarly, cumulative radiation exposure is currently a serious public health concern in Fukushima [3].

While air dose levels decrease rapidly due to the decay of short half-life radioactive materials and the weathering process in most radiation-contaminated areas [4], chronic internal radiation exposure accounts for a substantial fraction of the cumulative, long-term radiation exposure among residents in radiation-contaminated areas. This is largely due to the sustained radio-contamination of locally grown produce, as in the case of the Chernobyl disaster [5]. Reduction of chronic internal contaminations is a great public health concern in the affected areas, and several preventive measures, including thorough radiation inspection of food before shipment, public education, and individual dose monitoring, are warranted for prolonged periods after a nuclear disaster [6]. However, the efficacy of these measures for the reduction of individual internal contamination has not yet been investigated.

**Figure 1:**
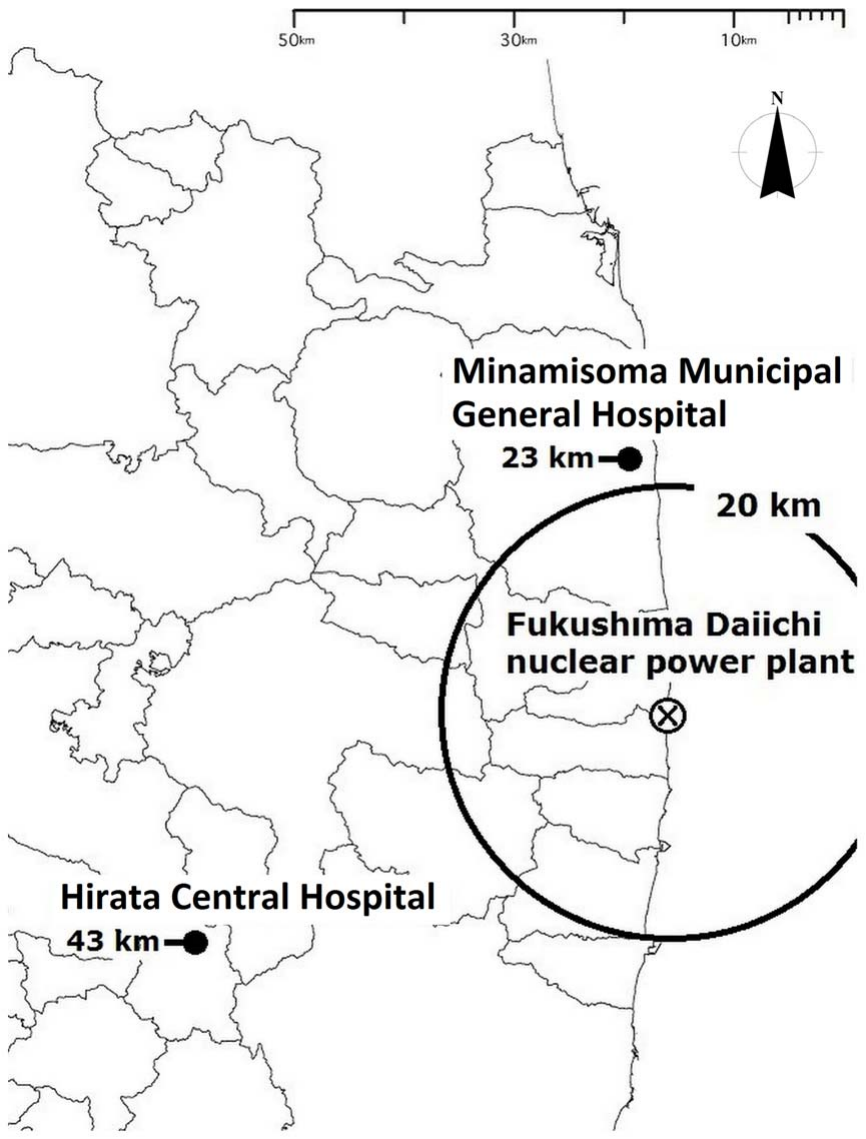
Geographical location of Minamisoma city, Minamisoma Municipal General Hospital, and Hirata Central Hospital, in relation to the Fukushima Daiichi nuclear power plant.

Levels of acute internal contamination immediately after the Fukushima Daiichi nuclear power plant disaster were minimal among the majority of residents in the affected areas [7]. Minamisoma is a coastal city located 14–38 km north of the Fukushima Daiichi nuclear plant, and was one of the most radiation-contaminated regions, with three different evacuation zones declared by the Japanese government on March 25, 2011 [8]. (Figure 1) Our initial screening in Minamisoma city showed that levels of internal contamination were below the whole body counter (WBC) detection limits among 65% of the screened residents [9]. Similar results were reported from screening at the Hirata Central Hospital in Fukushima, involving residents from all parts of the Fukushima prefecture [10], [11]. A subsequent study demonstrated that the levels of chronic internal contamination in children were diminishing in Minamisoma residents one year after the nuclear accident [12].

Conversely, other studies have showed high levels of internal contamination with radioactive cesium (Cs) in certain residents from the same area [10]. Levels of internal radioactive cesium in these cases occasionally reached over 100 Bq/kg, which was comparable to levels observed after the Chernobyl disaster [13]. It is important to implement targeted countermeasures for residents with high levels of internal contamination as indicated by the International Commission on Radiation Protection [14]. However, little information is currently available regarding the efficacy of individual interventions by medical care providers to reduce chronic internal contamination.

To attempt to reduce the high levels of internal contamination, we here performed careful dietary interventions in residents with high levels of internal contamination, with identification of suspected contaminated foods and regular checkups in our facilities, and evaluated the effectiveness of the intervention. Detailed description of these cases provides useful information regarding the high-risk behaviors associated with increased internal contamination, and regarding the precautions and life-style adjustments necessary for avoiding further increases in the levels of internal radiation exposure in people living in radiation-contaminated areas.

## Materials and Methods

### Basic characteristics of participants

Two hospitals in the Fukushima prefecture, which operate the voluntary internal radiation exposure screening programs, participated in this study: Minamisoma Municipal General Hospital and Hirata Central Hospital (Figure 1). A total of 30,622 individuals participated in the screening program from March 11, 2012 to March 10, 2013: 9,963 from Minamisoma Municipal General Hospital and 20,659 from Hirata Central Hospital. The study participants consisted of 14,397 men and 16,225 women. The median age was 14 years (range: 2–97 years). According to the checkup results published by the Fukushima prefectural government, these examinations accounted for approximately 20% of all examinations performed cooperatively with the Fukushima prefectural government in the corresponding period [15].

These two hospitals were the first two hospitals in Fukushima prefecture to implement independent, voluntary screening programs for internal radiation exposure after the Fukushima Daiichi nuclear disaster without the support of the Japanese national or Fukushima prefectural governments. Minamisoma Municipal General Hospital launched the screening program from July 2011, free of charge for all Minamisoma residents aged six and above. A program notification was sent to each household, including former residents who had evacuated elsewhere but could be tracked using the city's family registry. The notification was also disseminated on the hospital website and the city's public relations magazine. This city had a population of 72,000 before the earthquake, which dropped to approximately 10,000 in April 2011, and recovered to 46,720 in October 2013 [16]. The screening results from this hospital for the first year after the disaster have been published in a previous study [9]. Hirata Central Hospital, located 45 km southwest of the nuclear plant, operated an extensive screening program for people aged four and above from October 2011. The program included free local government-led checkups in the central district of Fukushima prefecture (Naka-dori) and in the neighboring prefecture (Ushiku city, Ibaragi), and voluntary participation of residents in the Fukushima prefecture without charge. Part of the data obtained from Hirata Central Hospital have been employed in a previous study by Hayano et al. in 2013 [10].

### Design and setting

Residents with internal Cs-137 contamination of more than 50 Bq/kg at the time of examination were included in this study. The participants were interviewed about their dietary preference at the outpatient clinic, and were advised to consume distributed food mainly and to refrain from consuming potentially contaminated foods without radiation inspection and local produces under shipment restrictions such as mushrooms, mountain vegetables, and meat of wild life. Three months after the interview, they were invited to follow-up examinations, and their dietary behaviors were re-assessed. Essentially, these subjects were offered pre- and post-test counseling for radiation risk behavior at first attendance, and again at a confirmatory follow-up examination. The short biological half-life of Cs-137 (approximately 100 days) means that quite large reductions in internal contamination can be observed in the time of the study if all chronic sources of contamination are attenuated.

### Pre-/post-test dietary counseling and interventions

Participants received medical examinations using unstructured interviews. A medical doctor asked whether respondents frequently consumed locally grown food produce without radiation inspection. A maximum allowed limit of 100 Bq/kg of Cs for general foods came into force on April 1, 2012 in Japan, and for this study, food from supermarkets was considered monitored regardless of its origin (Fukushima or non-Fukushima) [17]. Respondents were also asked whether they used tap water for drinking and cooking. They were advised to refrain from consuming potentially highly contaminated unmonitored foods, such as outdoor-grown mushrooms, mountain vegetables, and game meat (such as deer and wild boar, which are popular game foods in northern Japan). To confirm their contamination risk, samples of these foods were obtained from participants, and were examined for contamination levels. For the Cs measurements, the samples were prepared under routine food monitoring processes and assessed for Cs concentration (Standard Electrode Coaxial Ge Detector, Model: GC2018, Canberra Inc., Meriden, USA).

### Statistical analysis

To properly assess the impact of the dietary intervention, we examined heterogeneity of the radiation reduction rate of the radionuclides amongst the study subjects. Significant variability in the reduction rates may indicate heterogeneous influence of the intervention on subjects due to their varying adherence to the dietary advice, or unobserved factors, such as, genetic difference in metabolic response to the radioactive materials. Therefore, we first constructed a linear regression model on the reduction rate and estimated subject-specific unexplained effects on the rate adjusting for confounders, which were referred to as residuals. The detection of the heteroskedasticity in the residuals indicates that the reduction rate were significantly influenced by unobserved factors and we could not appropriately evaluate the effectiveness of the dietary intervention. We used the reduction rates of Cs-137 amount between 1st and 2nd measurement time as dependent variable, and age, sex, hospital, logarithm of days between the 1st and 2nd measurement dates as independent variables. Then, the heteroskedasticity of the residuals were tested using the White and Breusch-Pagan tests.

### Whole body counting and internal dose calculation

Minamisoma Municipal General Hospital and Hirata Central Hospital employ a whole body counter (WBC) system (Fastscan Model 2251, Canberra Inc., Meriden, USA). Detection limits of the system were set to 220 Bq for Cs-134 and 250 Bq for Cs-137 in Minamisoma Municipal General Hospital, and 300 Bq for both Cs-134 and Cs-137 in Hirata Central Hospital with a two-minute scan. The different detection limits between the hospitals were due to the difference in background air dose levels. Calibration of the machine was performed routinely according to the manufacturer's instructions [18]. Annual effective doses from internal contamination of ingested Cs-134 and Cs-137 were evaluated on the basis of the effective dose coefficients 1.9×10^−5^ and 1.3×10^−5^ mSv/Bq, respectively, derived from the International Commission on Radiological Protection, Publication 67, assuming that the amount of Cs activity detected at the WBC examinations was in an equilibrium state between consecutive ingestion and excretion throughout one year [19].

### Ethics approval

The study was approved by the institutional review board of the University of Tokyo (authorization number 25-40-1011). Written informed consent was obtained from participants or the next of kin, caretakers, or guardians on behalf of the minors/children enrolled in this study.

## Results

### Internal radiation exposure

**Figure 2:**
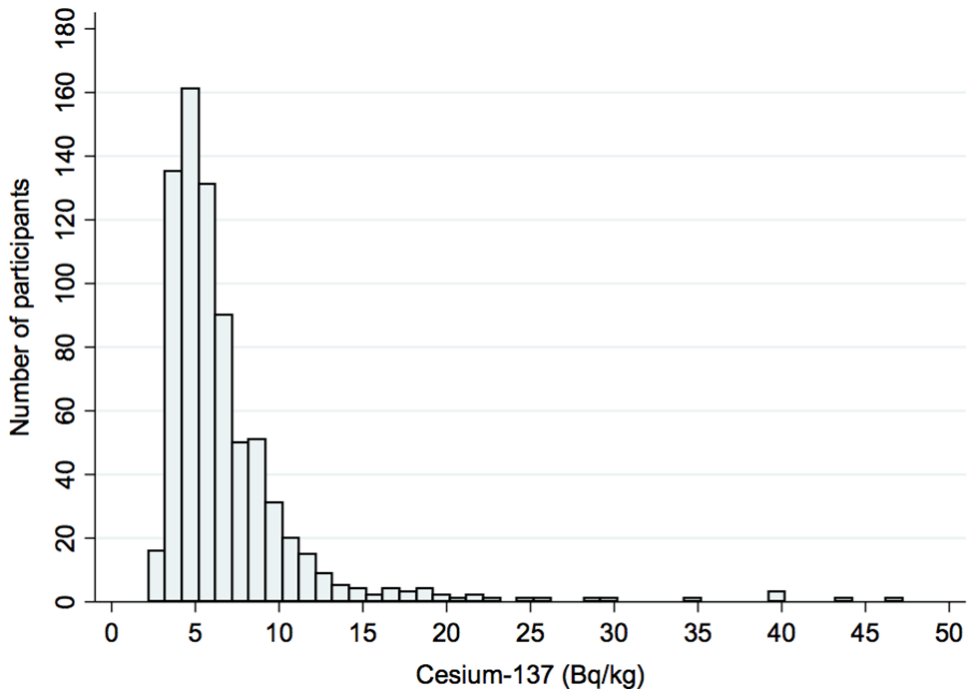
Distribution of Cs-137 (Bq/kg) in the analyzed population (less than 50Bq/kg).

Cs-137 was detected in 756 of 30,622 participants (2.5%): 612 from Minamisoma Municipal General Hospital (6.1%) and 144 from Hirata Central Hospital (0.7%). Distribution of Cs-137 [Bq/kg] is shown in Figure 2 for participants with concentration below 50 kg/bq. Nine residents exceeded 50 Bq/kg: three from Minamisoma Municipal General Hospital and six from Hirata Central Hospital (Table 1). We experimentally set the cut-off level of Cs-137 internal contamination to 50 Bq/kg in this study, since these residents showed unusually high levels of internal contamination compared to the rest of the residents in our clinics as was shown in Figure 2. None of these participants were involved in cleanup/decontamination work or reconstruction work in highly radio-contaminated areas. Three of the nine subjects were female, and the median age was 70 (range: 60–74 years). The median level of internal Cs-137 contamination at the initial screening was 4,830 Bq/body (range: 2,130–15,918 Bq/body), and 69.6 Bq/kg (range: 50.7–216.3 Bq/kg). The median annual effective dose estimated from the Cs-134 and Cs-137 burden was 0.30 mSv/year (range: 0.14– 0.97 mSv/year). There were three married couples amongst the nine residents. The measured levels of Cs-137 in the husbands and wives of these three couples were 111.6 and 69.6, 183.7 and 69.4, and 56.7 and 50.7 Bq/kg, respectively. All the husbands had a higher concentration than their wives.

**Table 1:** Results of repeated measurement among residents (n = 9) with internal Cs-137 burdens of more than 50 Bq/kg.

Patient							Cs-134		Cs-137			Cs-134		Cs-137			Cs-134		Cs-137	
No	Age	Sex	Hospital	Resident of	Family	1st measurement, date	Bq/body	Bq/kg	Bq/body	Bq/kg	2nd measurement, date	Bq/body	Bq/kg	Bq/body	Bq/kg	3rd measurement, date	Bq/body	Bq/kg	Bq/body	Bq/kg
1	70	M	Hirata	Kawamata	Family 1	9^th^ July, 2012	4160	66.0	7032	111.6	8^th^ Nov, 2012	1313	20.9	2547	40.6	19^th^ Feb, 2013	631	10.0	1069	16.9
2	66	F	Hirata	Kawamata	Family 1	13^th^ July, 2012	2471	40.0	4300	69.6	8^th^ Nov, 2012	695	11.2	1485	23.9	19^th^ Feb, 2013	ND*	ND	585	9.4
3	71	M	Minamisoma	Minamisoma	-	11^th^ July, 2012	6713	88.3	10730	141.2	6^th^ Nov, 2012	3288	43.8	5556	74.1	23^rd^ Apr, 2013	1717	21.2	3445	42.5
4	64	M	Hirata	Tamura	-	6^th^ Sep, 2012	9114	123.8	15918	216.3	6^th^ Dec, 2012	4122	56.0	7670	104.2	-	-	-	-	-
5	74	M	Hirata	Nihonmatsu	Family 2	2^nd^ Aug, 2012	7237	108.3	12270	183.7	8^th^ Nov, 2012	3204	47.7	6177	91.9	14^th^ Feb, 2013	1679	25.0	3600	53.7
6	74	F	Hirata	Nihonmatsu	Family 2	2^nd^ Aug, 2012	2894	41.6	4830	69.4	11^th^ Nov, 2012	1133	16.0	2139	30.3	4^th^ Feb, 2013	418	5.8	919	12.8
7	60	M	Hirata	Koriyama	-	15^th^ Apr, 2012	2203	42.6	3190	61.7	-	-	-	-	-	-	-	-	-	-
8	73	M	Minamisoma	Minamisoma	Family 3	16^th^ May, 2012	2090	36.7	3230	56.7	14^th^ Aug, 2012	1043	18.3	1695	29.7	19^th^ Feb, 2013	ND	ND	582	10.2
9	69	F	Minamisoma	Minamisoma	Family 3	16^th^ May, 2012	1442	34.3	2130	50.7	14^th^ Aug, 2012	466	11.1	711	16.9	19^th^ Feb, 2013	ND	ND	ND	ND

* ND represents not detected.

Detection limits: 220 Bq for Cs-134 and 250 Bq for Cs-137 in Minamisoma Municipal General Hospital, and 300 Bq for both Cs-134 and Cs-137 in Hirata Central Hospital.

Table 2 shows the results of assessment of produce grown locally without radiation inspection. One of the nine residents (subject number 7) did not answer questions about dietary preference. During the interview, it was revealed that all eight residents with available data consumed rice and milk after radiation inspection. Regarding water consumption, seven residents used tap water, one used bottled water, and one used well water with radiation inspection. However, all eight residents consumed homegrown vegetables without any radiation contamination monitoring, and often collected mushrooms in the wild or ate mushrooms cultivated on bed-logs in their homes. Table 3 shows detailed information on local food produce consumed without radiation inspection. Mushrooms were mainly outdoor-grown shiitake mushrooms. Accurate information as to the frequency or amount of the intake of the food was not available. The resident with the highest levels of Cs contamination in this study (subject number 4) reported regularly eating wild brawn meat and river fish, including mountain, rock, and rainbow trout without any radiation inspection, in addition to untested mushrooms and vegetables.

**Table 2:** Assessment of intake of local grown produce without radioactive inspection.

Patient No	Rice	Meat	Fish	Vegetables	Fruits	Mushrooms	Milk	Water
1	-	-	-	○	○	○	-	Tap water
2	-	-	-	○	○	○	-	Tap water
3	-	-	-	○	-	○	-	Tap water
4	-	○	○	○	-	○	-	Well water
5	-	-	-	○	○	○	-	Tap water
6	-	-	-	○	○	○	-	Tap water
7	N/A*	N/A	N/A	N/A	N/A	N/A	N/A	Mineral water
8	-	-	-	○	○	○	-	Tap water
9	-	-	-	○	○	○	-	Tap water

* N/A represents not applicable.

** ○ indicates that the respondent consumed this food without radiation inspection.

**Table 3:** Detailed information on local food produce comsumed without radioactive inspection.

Patient No	Vegetables	Mushrooms	Fruits	Meat	Fish
1	Garlic, bamboo shoots, chinese chive	Shiitake mushrooms	Dried persimmon	-	-
2	Same as Patient 1				
3	Aralia sprouts	Shiitake mushrooms	-	-	-
4	Radish, bamboo shoot, soybean, butterbur, bracken, aralia sprout, pumpkin, japanese ginger	Matsutake, sarcodon, and shiitake mushrooms	-	Game	Mountain, rock, and rainbow trout
5	Cucumber, tomato, chestnuts, bracken, torreya nuts, watermelon, aralia sprout, butterbur	Shiitake mushrooms	Dried persimmon	-	-
6	Same as Patient 5				
7	N/A*	N/A	N/A	N/A	N/A
8	Potatoes, green onions, japanese basil, ginkgo nuts, radishes	Shiitake mushrooms	Persimmon	-	-
9	Same as Patient 8				

* N/A represents not applicable.

### Results of the food measurement

The results of measurements of the regularly consumed foods are shown in Table 4. Cs was highly concentrated in dry mushrooms and wild brawn meat.

**Table 4:** Food measurement results.

	Date of inspection	Cs-134 (Bq/kg)	Cs-137 (Bq/kg)	Total Cs (Bq/kg)
Patients 1 and 2				
Shiitake mushroom	July, 2012	4,160	6,606	10,766
Chinese chive	Aug, 2012	59	94	153
				
Patient 4				
Game	Dec, 2012	453	793	1,246
Japanese ginger	Sep, 2012	ND (<5.2)*	ND (<6.6)	ND
Mountain trout	Sep, 2012	66	94	160
Rock trout	Sep, 2012	51	74	124
Rainbow trout	Sep, 2012	266	426	692
				
Patients 5 and 6				
Bracken	Aug, 2012	150	226	377
Dried persimmon	Aug, 2012	25	48	73
Dried shiitake mushroom	Aug, 2012	52,154	89,980	142,134
Chestnuts	Aug, 2012	302	489	791
Torreya nuts	Aug, 2012	388	613	1001
Shiitake mushroom	Feb, 2013	7,724	15,809	23,533

* ND represents ‘not detected’. The detection limit is presented in brackets.

The data on mushrooms from patient 4 was unavailable, since he had already consumed them.

### Results of the follow-up examinations

We advised all subjects to refrain from eating locally produced food without radiation inspection, and to consume food mainly from supermarkets or other food markets. During their second interviews, approximately three months after their initial radiation screening, seven of the eight residents mentioned that they were primarily purchasing and consuming rice, vegetables, mushrooms, and fruits from supermarkets, and consumed locally grown produce only after radiation inspection. However, one participant (number 4) described that while he quit eating wild mushrooms, game, and trout with high-levels of radio-contamination, he was still consuming vegetables from his garden. Re-examination of Cs levels showed remarkable reduction (almost half) in all of the nine residents (Table 1).

As for the heterogeneity in the subjects' radiation reduction rates of Cs-137, a regression analysis showed no dependence of the reduction rate on variables including age, sex and logarithm of days between the 1st and 2nd measurement dates (data were not shown). As no significant result was seen by the White and Breusch-Pagan tests (p = 0.3 and p = 0.8, respectively), we assume that the heteroskedasticity of the residuals does not affect our interpretation of the findings. This means that a homogeneous intervention effect might be robust across the study participants.

## Discussion

The maintenance of low levels of chronic internal radiation contamination among residents in radiation-contaminated areas after a nuclear disaster is a great public health concern, since a substantial fraction of cumulative radiation exposure is accounted for by chronic internal radiation exposure due to intake of locally grown produce with sustained radio-contamination.

Of note, only nine residents (0.03%) showed internal contamination of Cs-137 above 50 Bq/kg in this study. This suggests that levels of internal radiation contamination are minimal amongst most residents of the affected areas in Fukushima, and that preventive food control measures are sufficient to avoid further internal radiation contamination in Fukushima [20]. The majority of inhabitants in the area appear to be consuming agricultural products classified as safe under the food monitoring system, and/or accessing foods from non-contaminated areas through modern food distribution systems.

However, as mentioned, the present study showed that internal contamination was far higher in a small proportion of the residents. Nine residents exceeded internal contamination of Cs-137 above 50 Bq/kg. Clinicians need to be aware that even under seemingly successful food regulation regimes, some people will unknowingly consume highly contaminated foodstuffs and incur high levels of internal contamination. It is important to understand the high-risk internal contamination behaviors arising through daily lifestyle choices in radiation-contaminated areas. Although current preventive food control measures are sufficient, they should be maintained in the future.

Although it remains unknown whether the high levels of internal contamination in these residents were from chronic ingestion of contaminated food or from acute intake of radioactive materials immediately after the disaster, this study revealed that there may be similarities in dietary preferences amongst residents who have relatively high internal contamination levels, with these residents commonly consuming untested foodstuffs that have a high risk of radio-contamination, such as shiitake mushrooms, agricultural products, and wild animals and fish. This has been previously reported in studies after the Chernobyl accident [21]. However, it should be noted that the contamination levels vary among foodstuffs, and intake of local products in highly contaminated sites does not necessarily result in high levels of internal contamination [22]. Locally grown vegetables were consumed by all participants; however, agricultural products such as potatoes, leafy vegetables, and root vegetables, generally absorb low levels of radiation-contamination as a result of their low efficiency in transferring radioactive materials from the soil to the plant [23]. One of the most important mechanisms to reduce the risk of chronic internal radiation contamination is not to avoid intake of local products, but rather to recognize potentially highly contaminated foodstuffs, and to measure their Cs levels. Most commercial foodstuffs can be measured and restricted before entering the market, but it is quite difficult to regulate wild plant and animal products with high levels of contamination. Therefore, education activities aimed towards individuals who consume such untested foodstuffs, as exemplified by this study, are necessary.

While the present study indicate that it is especially important to avoid intake of locally grown produce with high levels of contamination, the most important method for prevention of chronic internal radiation exposure in the future is adequate food management, since the formation of radiation “hot-spots” by accumulation of radionuclides is unpredictable in affected agricultural areas. To further assess and reduce the long-term health risks of radiation in such areas, long-term measures, including continuous monitoring of internal contamination by WBCs, as well as of agricultural products, are essential.

This study indicated that dietary advice from medical care providers regarding the consumption of foods with low levels of contamination can change the dietary preferences of residents in high-risk areas, and subsequently result in a reduction of internal Cs contamination levels. The types and production sites of food need to be carefully considered when assessing the contamination risk, rather than simply being based on the background exposure levels of the settlement areas.

### Limitations of the study

The current screening program had a possible selection bias arising from the voluntary nature of the ongoing internal contamination monitoring system. Since the individuals who care most for their level of internal radiation exposure are more likely to participate in the screening program, the number of residents who had relatively high contamination levels (i.e., internal Cs-137 burden of more than 50 Bq/kg) might be underestimated. This may moreover result in an overestimation of the effect of dietary interventions since individuals who care about their diets and health are more likely to change their lifestyle based on medical advice. In addition, since no statistical analyses on the effects of the dietary interventions and no adjustment for other possible factors that might influence residents' dietary preference are currently available, our suggestions cannot go beyond speculation. However, we believe that our study makes important points about the management of radiation-release incidents, which occur very rarely and only in very specific locations. The lessons learned about controlling internal radiation exposure will be valuable for better managing the aftermath of similar incidents in the future.

## Conclusion

The present study found that a pre-/post-test counseling-based dietary intervention was effective for reducing internal contamination in residents near the damaged nuclear plant in Fukushima who showed high levels of internal contamination.
